# Supplementary material for: The interactome of intact mitochondria by cross-linking mass spectrometry provides evidence for coexisting respiratory supercomplexes
Source: Mol Cell Proteomics. 2017 Dec 8;17(2):216–32. doi: 10.1074/mcp.RA117.000470 (PMC5795388; doi:10.1074/mcp.RA117.000470)
Supplement: Supplemental Data [file supp_RA117.000470_133922_0_supp_23978_fzffwf.zip › spectra_annotation/mito_spectra_annotation/491-1-3-1-1-1.pdf]

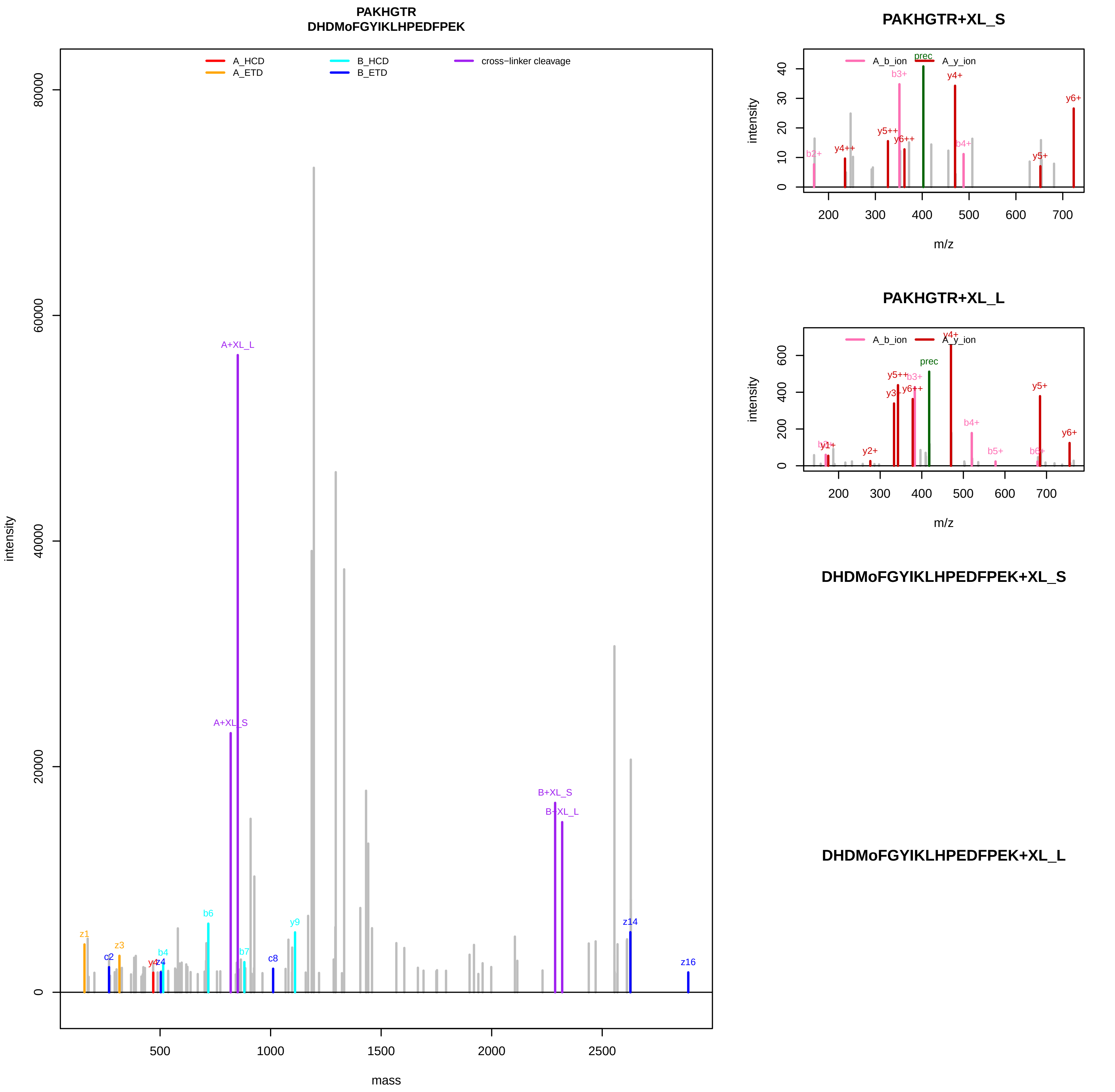
PAKHGTR
DHDMoFGYIKLHPEDFPEK
A_HCD
A_ETD
B_HCD
B_ETD
cross−linker cleavage
A+XL_L
A+XL_S
B+XL_S
B+XL_L
z1
z3
c2
y4
z4
b4
b6
b7
c8
y9
z14
z16
intensity
mass
0
20000
40000
60000
80000
500
1000
1500
2000
2500
PAKHGTR+XL_S
A_b_ion
A_y_ion
prec
b2+
y4++
y5++
b3+
y6++
y4+
b4+
y5+
y6+
m/z
PAKHGTR+XL_L
y1+
y2+
y3+
y5++
b3+
y6++
b4+
b5+
b6+
DHDMoFGYIKLHPEDFPEK+XL_S
DHDMoFGYIKLHPEDFPEK+XL_L
